# A Rare Case of Spontaneous Splenic Rupture as the Index Presentation of Chronic Myeloid Leukemia

**DOI:** 10.7759/cureus.19647

**Published:** 2021-11-16

**Authors:** Sri Hari Priya Vemulakonda, Sree Subramaniyan S, Ankit Jain, Abhinaya Reddy, Vishnu Prasad Nelamangala Ramakrishnaiah

**Affiliations:** 1 General Surgery, Jawaharlal Institute of Postgraduate Medical Education and Research, Puducherry, IND

**Keywords:** chronic myeloid leukemia (cml), spontaneous splenic rupture, total splenectomy

## Abstract

Splenic injury is usually caused by blunt trauma to the abdomen. Very rarely, spontaneous rupture can occur in patients with splenomegaly due to various underlying pathological conditions such as hematological, neoplastic, inflammatory, and infectious diseases. Here, we report the case of a 48-year-old gentleman who presented to the emergency department with sudden-onset pain in the abdomen and hypotension. Blood investigation revealed anemia and leukocytosis with blast cells on peripheral smear, suggestive of chronic myeloid leukemia (CML) in the chronic phase. Contrast-enhanced computed tomography revealed splenomegaly with grade three splenic laceration and a subcapsular hematoma with hemoperitoneum. Because of persistent hemodynamic instability, despite resuscitation, he underwent emergency splenectomy. The postoperative period was uneventful. Bone marrow biopsy revealed CML in the chronic phase with World Health Organization grade I reticulin fibrosis. Subsequently, he was started on hydroxyurea and discharged for further follow-up with medical oncology.

## Introduction

Spontaneous splenic rupture (SSR) is a rare clinical entity, with life-threatening complications [1]. Prompt diagnosis and treatment are required to ensure patient survival. SSR is atraumatic and occurs in the pathologically enlarged spleen due to various underlying causes, either benign or neoplastic [2]. Hematological malignancies such as acute leukemia or lymphomas as the underlying cause of SSR are rare. Here, we present a rare case of SSR as the index presentation of chronic myeloid leukemia (CML).

## Case presentation

A 48-year-old gentleman, a known case of rheumatic heart disease with mitral valve regurgitation and bicuspid aortic valve, presented to the emergency department complaining of pain in the left upper abdomen for two days. The pain was sudden in onset, progressive, not responding to analgesics, and associated with abdominal distension. There was no history of nausea, vomiting, fever, and constipation. Additionally, there was no history of trauma or any surgery. On presentation, he was conscious and oriented, with tachycardia (120 beats/minutes) and hypotension (90/60 mmHg). On examination, his abdomen was distended with tenderness in the left hypochondriac region.

**Figure 1:**
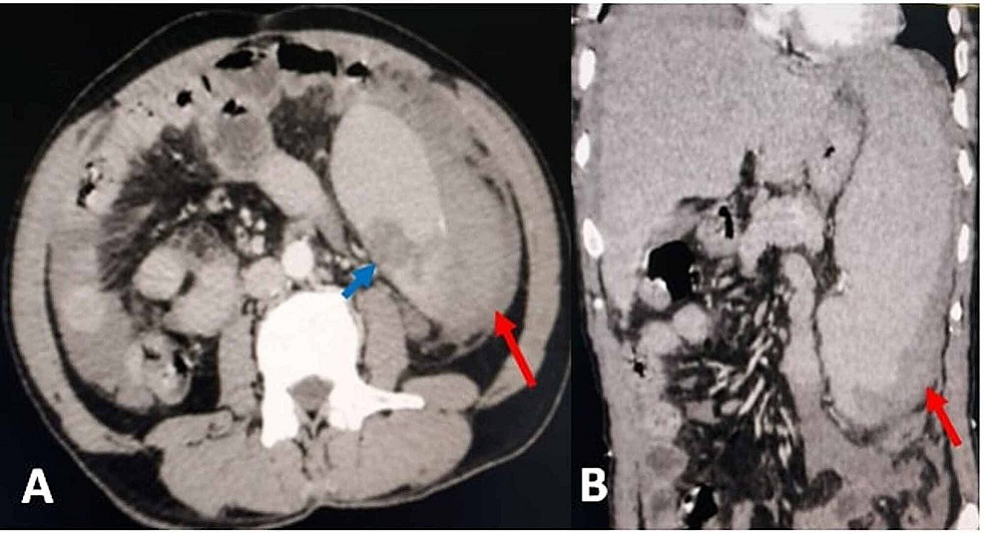
CECT showing a grade three splenic laceration 3.3 × 2.6 cm in the lower pole (blue arrow) and subcapsular hematoma involving >50% of the surface area (red arrow) with hemoperitoneum. A: axial section; B: coronal section CECT: contrast-enhanced computed tomography

Blood investigation showed normocytic normochromic anemia (hemoglobin 6.5 g/dL) and a total leucocyte count of 1.94 lakhs/dL. Peripheral smear revealed 3% blasts cells, myeloid bulge, and basophilia, suggestive of CML in the chronic phase. Ultrasonography of the abdomen revealed free fluid abdomen, splenomegaly, and a subcapsular splenic hematoma. Contrast-enhanced computed tomography (CECT) confirmed splenomegaly (19 cm) with a grade three splenic laceration (3.3 × 2.6 cm) in the lower pole with a subcapsular hematoma (>50% of surface area) with hemoperitoneum (Figure 1).

**Figure 2:**
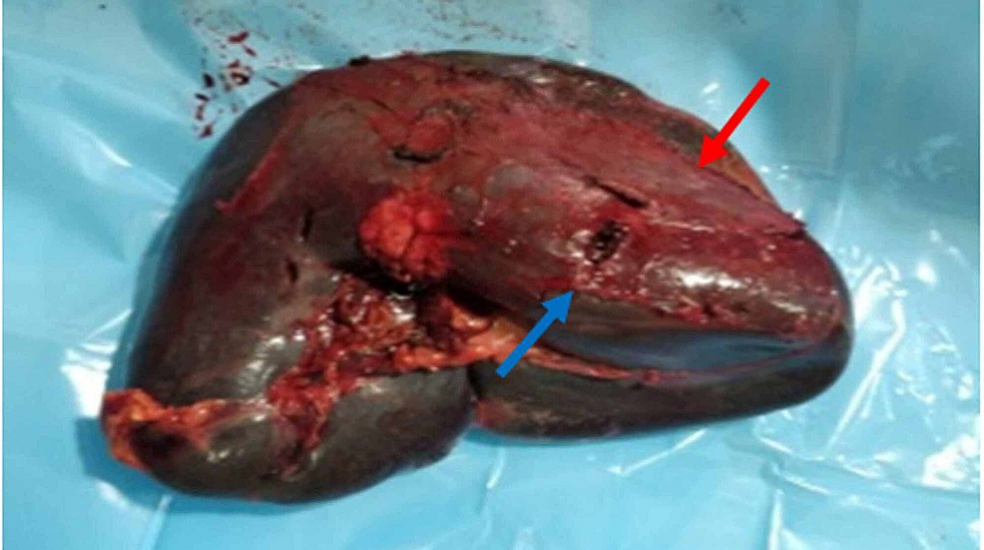
Gross specimen: a 3 cm laceration in the lower pole (blue arrow) with a ruptured subcapsular hematoma (red arrow).

Because of hemodynamic instability, despite resuscitation, he underwent emergency splenectomy. Intraoperatively, 1,200 mL of hemoperitoneum was observed. Spleen was enlarged (18 cm) with a 3 cm laceration in the lower pole and a ruptured subcapsular hematoma (Figure 2).

**Figure 3:**
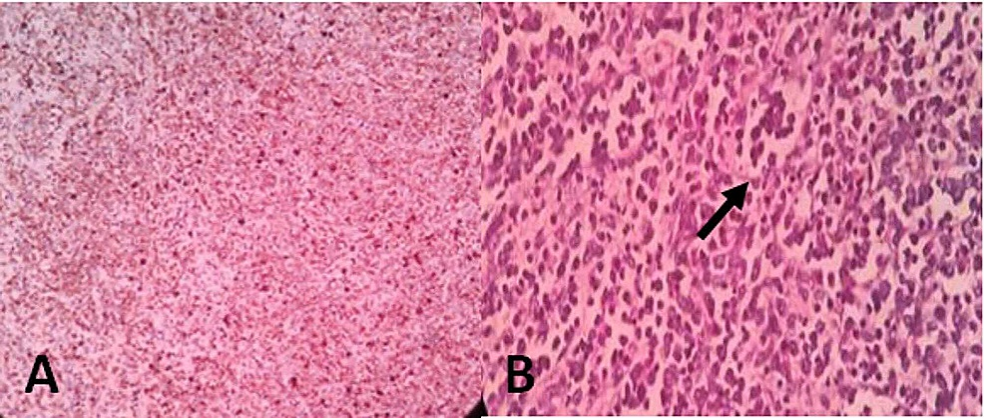
Biopsy from the spleen showing myeloid blast cells (A) and megakaryocyte (B) (black arrow).

Intraoperatively, he was transfused with four units of packed cells, fresh frozen plasma, and platelets. The postoperative period was uneventful. He was further evaluated for CML with bone marrow biopsy, which revealed CML in the chronic phase with World Health Organization grade I reticulin fibrosis. Histopathological examination of the spleen revealed features consistent with CML in the chronic phase (Figure 3).

He was started on hydroxyurea and discharged for further follow-up with medical oncology.

## Discussion

Atraumatic splenic rupture or SSR was described for the first time in 1924 [1]. Atraumatic pathological splenic rupture occurs in patients with underlying pathological conditions and histopathological changes within the spleen [2]. It occurs in patients with splenomegaly due to various underlying pathological conditions, such as (i) neoplastic (30.3%): hematological such as chronic lymphocytic leukemia, Hodgkin’s lymphoma, CML, and non-hematological such as angiosarcoma; (ii) infectious diseases (27.3%) such as malaria, mononucleosis, and other viral infections; (iii) inflammatory disorders (20%) such as pancreatitis, abscess, amyloidosis; and (iv) drugs and chemotherapy such as filgrastim (6.8%) [2]. Very few cases (7%) have been described in patients with a normal spleen, termed atraumatic idiopathic splenic rupture [2].

CML is a myeloproliferative neoplasm with reciprocal translocation of *ABL* on chromosome 9 to the *BCR* gene region, forming the Philadelphia chromosome which involves all hemopoietic cell lineages except mature T-cells. Its annual incidence ranges from 120,000 to 200,000 cases [3]. CML presents as the following three phases: chronic, accelerated, and blast crisis. Most patients present in the chronic phase with anemia, malaise, and weight loss. Very rarely, CML patients can present with SSR (0.72%). The rupture mechanism has been hypothesized as the malignant cell infiltration of the splenic parenchyma, resulting in its volume exceeding the capacity of the non-distensible splenic capsule, which causes rupture [4]. Concomitant coagulation disorders, if present, likely lead to subscapular hemorrhage and splenic infarction.

The clinical diagnosis of SSR is challenging. Patients present with a history of spontaneous onset of acute abdominal pain usually radiating to the left shoulder (Kehr’s sign), in association with fever, nausea, or vomiting [4]. Rarely, there may be a history of trivial trauma. In patients with hematological conditions, abdominal pain is the most common symptom (95%) [5]. On examination, the patients have a varying degree of tachycardia, tachypnea, and hypotension. Blood investigations show low hemoglobin levels with other changes as per the underlying pathological condition causing splenomegaly if any. Ultrasound of the abdomen is the initial investigation that may show free fluid in the abdomen with splenomegaly. However, CECT is the investigation of choice for diagnosis as it can show the grading of the splenic injury in detail.

SSR, similar to traumatic injury, can be managed either surgically (splenectomy) or conservatively (clinical monitoring). If the patient is hemodynamically stable, conservative management can be attempted. However, compared to patients with blunt abdominal trauma, the rate of successful non-surgical management is low [6]. Of all the SSR cases reported in the literature, 86.5% of the patients underwent splenectomy [2,7]. Compared to traumatic rupture patients, pre-existing splenomegaly, underlying neoplastic disorders, and advanced age are the factors associated with an increased rate of non-surgical management failure. Angio-embolization can be attempted in patients with limited vascular injury planned for conservative management [4]. However, due to the rare incidence of SSR, no data are available regarding its success rate. For patients who are hemodynamically unstable at presentation or those who become unstable on conservative management, splenectomy is the treatment of choice. Splenorraphy and partial or total splenectomy may be performed [8]. SSR has a mortality rate of 12% and can go up to 21% in neoplastic disorders [3]. Therefore, SSR cases associated with neoplastic etiologies should undergo a total splenectomy [2]. In SSR patients without apparent neoplastic disorders, total splenectomy may be justified for four reasons. First, the histological examination of the spleen will help in the diagnosis of underlying systemic diseases. Second, there is a high incidence of malignancy in SSR. The involvement of the spleen by malignant cells will prohibit any organ-preserving approach. Third, there may be functional hyposplenism due to infiltration of the splenic parenchyma. Therefore, a splenectomy will not increase the risk of an overwhelming post-splenectomy infection. Fourth, a higher failure rate of non-surgical management in patients with SSR. Early surgical treatment increases patient survival by up to 60% [9].

## Conclusions

SSR is an important differential diagnosis for an acute abdomen, especially in patients with splenomegaly. In a case of atraumatic splenic rupture, similar to traumatic injury, prompt recognition and surgical intervention are the best means of definitive care. Given the relatively high failure rate for non-operative management, total splenectomy is the preferred surgical option.
